# Influenza virus vector iNS1 expressing bovine papillomavirus 1 (BPV1) antigens efficiently induces tumour regression in equine sarcoid patients

**DOI:** 10.1371/journal.pone.0260155

**Published:** 2021-11-19

**Authors:** Christoph Jindra, Edmund K. Hainisch, Andrea Rümmele, Markus Wolschek, Thomas Muster, Sabine Brandt

**Affiliations:** 1 Department of Companion Animals and Horses, Division of Equine Surgery, Research Group Oncology, University of Veterinary Medicine, Vienna, Austria; 2 BlueSky Immunotherapies, Vienna, Austria; Instituto Butantan, BRAZIL

## Abstract

Bovine papillomaviruses types 1 and 2 (BPV1, BPV2) commonly induce skin tumours termed sarcoids in horses and other equids. Sarcoids seriously compromise the health and welfare of affected individuals due to their propensity to resist treatment and reoccur in a more severe form. We have developed influenza (Flu) A and B virus vectors that harbour a truncated NS1 gene (iNS) assuring interferon induction and co-express shuffled BPV1 E6 and E7 antigens for sarcoid immunotherapy. In a safety trial involving 12 healthy horses, intradermal administration of iNSA/E6E7^equ^ and iNSB/E6E7^equ^ was well tolerated, with the only transient side effect being mild fever in four horses. Repeated screening of secretions and faeces by RT-PCR and plaque assay revealed no virus shedding, thus also confirming biological safety. In a patient trial involving 29 horses bearing BPV1-induced single or multiple sarcoids, at least one lesion per horse was intratumourally injected and then boosted with iNSA/E6E7^equ^ and/or iNSB/E6E7^equ^. The treatment induced a systemic antitumour response as reflected by the synchronous regression of injected and non-injected lesions. Irrespective of vaccination schemes, complete tumour regression was achieved in 10/29 horses. In 10/29 horses, regression is still ongoing (May 2021). Intriguingly, scrapings collected from former tumour sites in two patients tested negative by BPV1 PCR. Nine severely affected individuals with a history of unsuccessful therapeutic attempts did not (6/29) or only transiently (3/29) respond to the treatment. INSA/E6E7^equ^ and iNSB/E6E7^equ^ proved safe and effective in significantly reducing the tumour burden even in severe cases.

## Introduction

Equine sarcoids are non-metastasising, yet locally aggressive cutaneous fibropapillomas induced by bovine delta-papillomaviruses of type 1, 2 and possibly type 13 (BPV1, BPV2, BPV13) [1, 2]. According to their clinical appearance, sarcoids are classified as mild-type occult or verrucous lesions, more aggressive nodular, fibroblastic or mixed tumours, or malevolent sarcoids invading local lymph nodes [3]. The high veterinary relevance of sarcoids is not only due to their exceptionally high incidence but also to their pronounced propensity to progress from mild single lesions to more severe multiple forms, especially upon accidental or iatrogenic trauma. Consequently, sarcoids still constitute the number one dermatological reason for euthanasia of affected equids although they do not metastasise [4]. Sarcoids are also economically relevant tumours in that they mainly develop in young adult equids, may compromise the use of affected animals as leisure, sport, breeding or working equids and pronouncedly reduce their market value [5]. The response of sarcoids to state-of-the-art treatment such as surgical excision, ligature, cryo- and chemotherapy or combinations of these is not satisfactory. In a substantial number of cases, treatment leads to recurrence of the disease, usually in a more aggressive form, rather than to tumour eradication. Therefore, there is a strong need for developing more effective sarcoid therapeutics [6–8].

Papillomaviruses (PVs) are small, non-enveloped viruses consisting of an icosahedral capsid harbouring a short (< 8 kbp) circular dsDNA genome. Whereas most PVs show a pronounced tropism for cutaneous or mucosal keratinocytes and are highly species-specific, δ-PVs do not adhere to this rule. They also or preferentially infect dermal fibroblasts, which may explain their wider host-range, comprising many ungulate species including equids, e.g. horses, donkeys, zebras or mules [1]. Genetic analyses have mapped the transforming activities of BPV1 and BPV2 to the early genes E5, E6 and E7 [9]. The respective oncoproteins are expressed throughout tumour development and act in a combined manner. They assure the survival of infected host cells by inducing their immortalisation, hyperproliferation and anoikis-independent growth [10–12]. In this context, the E6 oncoprotein acts as transcriptional activator, which abrogates the apoptotic and cell cycle arrest functions of the tumour suppressor protein p53 by downregulating CBP/p300 transcription. E6 also competitively binds to the focal adhesion protein paxillin, thus abrogating interaction of the latter with other adhesion proteins. This in turn allows for infected cells to grow in an anchorage-independent manner [13]. The E7 oncoprotein is co-involved in this process via binding to p600 [14]. The small hydrophobic E5 oncoprotein forms dimers and oligomers, and in this state, interacts with important host cell regulatory proteins, most notably the PDGFβ-R. Binding of E5 to this receptor leads to sustained activation of the latter–a prerequisite for E5-induced transformation of infected cells [15]. E5 also inhibits MHC class I transcription, expression and transport to the cell surface, thus allowing the virus to escape from immune surveillance [16]. While equine sarcoids are predominantly associated with BPV1 infection in Northern and Central Europe as well as in the Eastern USA, BPV2 prevails in sarcoids in the Western USA [17–21]. In 2013, a novel BPV termed BPV13 was identified in warts of Brazilian cattle. The genetic characterisation of BPV13 revealed a high similarity to BPV2 (>91%) and BPV1 (>87%) and an identical genomic organisation, suggesting that BPV13 possesses all the necessary features for inducing fibropapilloma [22]. This assumption was further corroborated by the detection of BPV13 DNA in equine sarcoids [2]. In addition to infection by BPV1, BPV2 and possibly BPV13, a host genetic susceptibility [23], and specifically expressed microRNAs–notably a large cluster on chromosome 24 [24]–likely contribute to sarcoid development.

The widely recognized association of BPV1 and BPV2 with sarcoid development raises the possibility of using therapeutic strategies targeting viral antigens. Whereas naturally acquired sarcoids usually persist and progress without or despite treatment, “pseudo-sarcoids” experimentally induced by wild-type BPV1 or BPV2 virions regress spontaneously due to an effective immune response [25–28]. Hence, re-instructing the locally compromised immune system to fight PV infection and related malignancies seems to be an attractive therapeutic concept. In contrast to prophylactic PV virus-like particle (VLP)-based vaccines that target the viral capsid [29], there is general agreement that therapeutic vaccines should be directed against early transforming and regulatory PV proteins [30].

As early as 1995, it was shown that vaccination of cattle with BPV4 E7 prior to challenge with wild-type (wt) virions retarded the development of papillomas and promoted their early regression. E7-specific T cells were detected at high levels shortly after challenge and three T cell epitopes were identified [31].

Considerable efforts have been made to translate this and similar findings obtained in animal PV models into human medicine. Various types of vaccines that mainly target the E6 and E7 oncoproteins are in development for the immunotherapy of high-risk human PV (hrHPV)-induced cancers. They include vector-based viral or bacterial systems as well as peptide-, protein-, DNA- and dendritic cell (DC)-based vaccines [30, 32, 33]. Promising systems targeting hrHPV16 E6 and/or E7 were shown to act via induction of immune responses mediated by CD4^+^ T helper type 1 cells (Th1) and CD8^+^ cytotoxic T lymphocytes (CTLs). These observations reflect the potential of E6 and E7 as therapeutic targets and the great importance of T cell responses, particularly CTL responses, for the induction of an immunotherapeutic effect [30, 32–34].

We have previously established human influenza (Flu) viruses lacking the C-terminal part of the non-structural gene NS1 (delNS) [35]. We showed that this deletion confers live attenuation because it fully compromises the interferon (IFN)-antagonising activity of NS1 required for the successful establishment of infection. As a result, the administration of Flu viruses expressing a truncated NS1 results in a strong IFN response in IFN-competent organisms [35–37]. The NS1 open reading frame (ORF) also proved suitable for the insertion of foreign gene sequences [38–41]. Prime and boost immunisation of rodents with different delNS virus serotypes proved effective in inducing a strong, transgene-specific T cell response [30, 38, 42]. Based on these data, we generated delNS influenza A and B viruses expressing mutation-inactivated HPV16 oncogenes E6 and E7 (delNSA/E6E7^hum^, delNSB/E6E7^hum^) as immunotherapeutic vaccine candidates for prime and boost vaccination. Importantly, intralesional injection of mice with established TC-1 tumours using these viruses induced complete tumour regression in 25% of all animals and significantly reduced tumour growth in 50% of mice [43–45].

To enhance antitumour properties, we then developed influenza A and B viruses harbouring an NS1 gene with a partial deletion (iNS1) and co-expressing shuffled BPV1 E6 and E7 antigens (iNSA/E6E7^equ^ and iNSB/E6E7^equ^) as potential sarcoid immunotherapeutics. The specific partial deletion resulted in iNS-based vectors, which induce high levels of IFN while–unlike the fully deleted NS1 deletion mutants–retaining the ability to replicate efficiently in IFN-sensitive tumour cells. In this context, we have previously shown that such partly deleted mutants have better antitumour properties and are less sensitive to the growth inhibitory effects of IFN [46]. In this article, we report on the generation of these viruses, their safety in tumour-free horses, and their therapeutic efficacy in sarcoid-affected equine patients.

## Materials and methods

### Generation of iNSA/E6E7^equ^ and iNSB/E6E7^equ^

**Fig 1:**
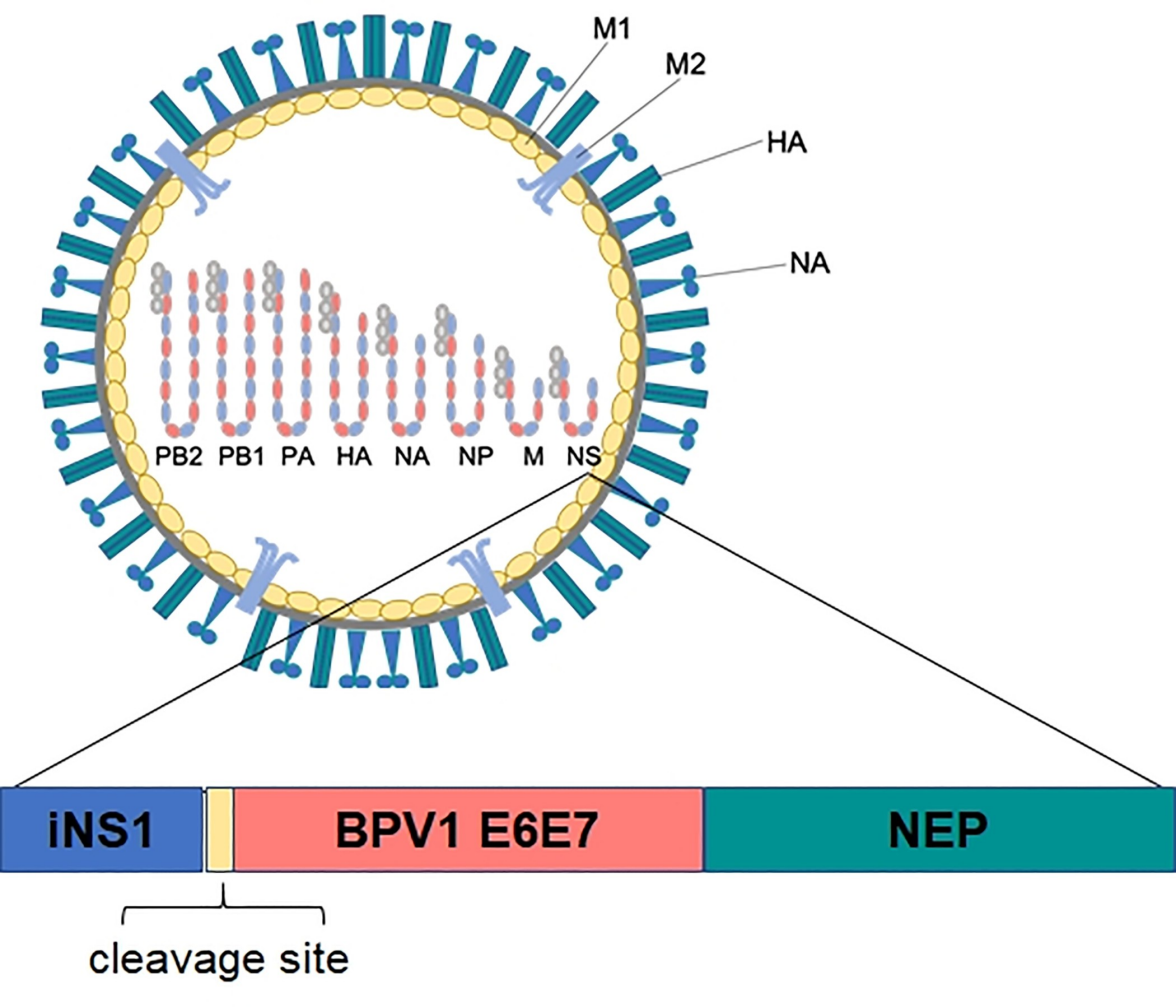
Schematics of iNSA or B/E6E7^equ^. The Flu A or B NS1 gene was truncated to the first 106 C-terminal codons (iNS1) and shuffled BPV1 E6 and E7 sequences were inserted into the NS1 ORF.

A shuffled BPV1 E6E7 fusion gene was designed and synthesised (GeneArt, ThermoFisher Scientific, Vienna, Austria). The fusion gene was sub-cloned into a pHW2000 plasmid derivative [47] containing a partially deleted NS1 gene leading to the expression of the first 106 C-terminal amino acids (iNS1) under the control of Pol I and Pol II promoters for vRNA and mRNA transcription (Fig 1). INS1 influenza A and B viruses containing the shuffled BPV1 E6E7 gene sequences were obtained by genetic engineering as previously described [43, 45] and termed iNSA/E6E7^equ^ and iNSB/E6E7^equ^. The internal segments of FluA were derived from the IVR-116 vaccine strain (WHO) that originated from A/Puerto Rico/8/34 for PA, PB2, NP, M, and A/Texas/1/77 for PB1. The viral HA and NA genes were derived from H1N1 virus A/New Caledonia/20/99. For FluB, all viral segments were derived from B/Thüringen/02/06 (B/Jiangsu/10/03-like). African green monkey kidney (Vero) cells [48] were seeded at a density of 80,000 cells/cm^2^ one day prior to electroporation and then co-transfected with 0.5 μg of plasmid pHW-ANS106-P2A-SP-E6E7shuffled or pHW-BNS106-P2A-SP-E6E7shuffled, expressing both vRNA and mRNA, and 0.5 μg of each bidirectional plasmid encoding the remaining seven segments using Nucleofector™ II technology (Lonza, Cologne, Germany). INSA/E6E7^equ^ and iNSB/E6E7^equ^ were collected with culture supernatant after cultivation for four to five days.

### Evaluation of transgene stability and expression

To verify transgene stability in the iNS1 reading frame, iNSA/E6E7^equ^ and iNSB/E6E7^equ^ were passaged in Vero cells for a minimum of five rounds. Total viral RNA was isolated using a QIAamp viral RNA mini kit (Qiagen, Hilden, Germany) according to manufacturer’s instructions and subjected to reverse transcription (RT) using uni-12 primer for Flu A (5’-AGCAAAAGCAGG-3’) and Uni-13 primer for Flu B (5’-AGCAGAAGCAGAG-3’) (Eurofins, Vienna, Austria) using a SuperScript® III First-Strand Synthesis System according to manufacturer’s instructions (Invitrogen, ThermoFisher Scientific). To amplify the iNSA-and iNSB-E6E7 regions, the following PCR primers were used: FluAf 5´-AGC AAA AGC AGG GTG ACA AAG-3’, FluAr 5´-CTC TTG CTC CAC TTC AAG C-3´ and FluBf 5´- AGC AGA AGC AGA GCA TTT G-3´, FluBr 5´-AGT AGT AAC AAG AGG ATT TTT A-3´ (Eurofins). PCR was carried out with GoTaq® DNA Polymerase following manufacturer’s recommendations (Promega, Vienna, Austria). Amplification products (16 μl) were electrophoretically separated in 1.5% TAE-gels and visualised by ethidium bromide staining.

For the analysis of transgene expression, equine primary palatal fibroblasts (eqPALF; kindly provided by Prof. Lubna Nasir, University of Glasgow, UK) were grown to sub-confluence for 12 hours, infected with iNSA/E6E7^equ^ or iNSB/E6E7^equ^ (10^5^ pfu) and then incubated for 12 hours. Cells were harvested, lysed in Laemmli-sample buffer, boiled for 5 minutes, and analysed by SDS-PAGE and Western blot. The antibodies used in these assays comprised mouse anti-influenza A nucleoprotein (NP) blend clone A1/A3 (1:5000; Millipore, Vienna, Austria) or mouse anti-influenza B NP clone B2 (Millipore); rabbit anti-BPV1 E6 and E7 antibodies (1:2000; a gift from Scott Vande Pol, University of Virginia, USA) and mouse anti-beta actin clone AC-74 (1:5000; Sigma-Aldrich). Proteins were separated on 10–15% gradient Tris-glycine SDS gels (Biorad) using the Tris-glycine SDS buffer system. Western blotting was performed by electrophoretic transfer of proteins to a polyvinyl difluoride (PVDF) membrane (Millipore). Bound antibodies on the blot were detected with HRP-conjugated goat anti-rabbit or goat anti-mouse antibodies (both from Biorad, Vienna, Austria; 1:10,000) using chemiluminescent substrates. Images were captured and analysed using a FluorChem FC3 system and its image analysis software (Biozym, Vienna, Austria).

### Assessment of virus concentrations

Vero cells were seeded in 96-well plates at a density of 50,000 cells/well 24 h before the concentration analysis. Cells were then infected with serial dilutions of iNSA/E6E7^equ^ or iNSB/E6E7^equ^ in the presence of 0.5 ng/ml amphotericin B (Sigma-Aldrich). After incubation for 20 hours at 37°C (FluA) or 33°C (FluB), cells were harvested, fixed in 4% formalin solution, permeabilized with 0.2% Triton^TM^ X-100 (Sigma-Aldrich) in PBS, stained with anti-influenza A NP or anti-influenza B NP (Biorad, Vienna, Austria) and anti-mouse IgG Alexa Fluor 488 conjugated (Invitrogen, ThermoFisher Scientific). Infected cells were counted on a Cytation Imaging Reader (BioTek, Bad Friedrichshall, Germany) and virus concentrations were calculated.

### Generation of high-titre vaccine batches

Vero cells were seeded at a density of 80,000 cells per cm^2^ one day before infection. For iNSA/E6E7^equ^, cells were infected at a moi of 0.1 in OptiPro SFM supplemented with 1% 10x TrypLE and 0.25 ng/ml amphotericin B (all from Sigma-Aldrich) and incubated at 37°C. The medium was substituted on day 1 after infection and the supernatant collected on day 3 after infection. For iNSB/E6E7^equ^, cells were infected at a moi of 0.02 in OptiPro SFM supplemented with 1% 10x TrypLE and 0.5 ng/ml amphotericin B, incubated at 33°C and the supernatant was collected on day three after infection. Supernatants were treated with 750U Benzonase Nuclease (Sigma-Aldrich) to reduce cellular DNA and influenza viruses were concentrated by polyethylene glycol (PEG) precipitation. Final viral concentrations ranged between 9.5 and 10.2 log FFU/ml.

### Safety trial

Twelve horses kindly provided by a cooperating farm were enrolled in the study and kept at the Biosafety Level 2 (BSL-2) horse facility (treatment phase) and then at the Vetfarm of the Vetmeduni Equine University Hospital (post-treatment mentoring phase) (Table 1). The horses met the following inclusion criteria: good general health condition, no clinical signs of sarcoid disease as assessed by clinical examination and no BPV1/2 infection as determined by routine BPV1/2 E5 PCR [49] from hair root DNA. To allow for intratumoural injections, transient pseudo-sarcoids were induced in all horses. To this aim, the horses were intradermally inoculated with cow wart-derived wt BPV1 virions on both sides of the neck (5 wheels per side à 1.2 x 10^5^ infectious virions in 100 μl PBS per injection) as described previously [25, 50]. Then the horses were monitored twice weekly and the tumour diameters were assessed using callipers. When all pseudo-sarcoids had reached diameters > 2 cm, lesions on the left side of the neck were injected in six horses with iNSA/E6E7^equ^ and iNSB/E6E7^equ^, and in six horses with virus carrier solution (SNH buffer: 25 mM HEPES, 200 mM sucrose, 100 mM NaCl adjusted to pH 7.5 by addition of NaOH) in a blinded approach. Lesions on the right side of the neck were left untouched. The vaccination scheme was the following: iNSA/E6E7^equ^ (500 μl à 9 log FFU) or SNH buffer on days 0, 2 and 4, and iNSB/E6E7^equ^ (500 μl à 9 log FFU) or SNH buffer on days 7, 9 and 11. All horses were closely monitored until all lesions had completely resolved, with special emphasis being put on local and systemic reactions attributable to intratumoural injections. Behavioural parameters (alert, bright and responsive), vital signs (body temperature, respiratory and heart rates) and local and systemic effects of injections were recorded twice daily until d15. Since no virus shedding was observed, all horses were subsequently moved from the BSL-2 facility to the Vetfarm providing ideal horse stabling conditions. Pseudo-sarcoid diameters were assessed twice a week until week ten, and then weekly using callipers. After all pseudo-sarcoids had completely resolved, the horses returned to their owner.

**Table 1:** Intratumoural injection with iNSA/E6E7^equ^ and iNSB/E6E7^equ^ is well tolerated.

Horse	Age	Sex	Breed	Maximum transient fever*	Oedema at injected tumour sites	Oedema at non-injected tumour sites
Active Substance Group						
FL04	13	m	WB	39.5	yes	Yes
FL05	14	m	Hucul	no fever	yes	Yes
FL06	7	g	Pony	38.6	yes	Yes
FL07	6	g	Haflinger	38.8	yes	Yes
FL08	4	g	Pony	no fever	yes	Yes
FL12	14	m	WB	38.4	yes	Yes
Placebo group						
FL01	9	g	WB	no fever	yes^§^	No
FL02	11	m	WB	no fever	no	No
FL03	15	g	Pony	no fever	no	No
FL09	12	g	WB	no fever	no	No
FL10	6	g	STB	no fever	no	No
FL11	9	m	STB	no fever	no	No

m: mare; g: gelding; WB: Warmblood horse; STB: Standardbred horse (Trotter).

* > 38°C; maximum duration < 24h, observed after first injection with iNSA/E6E7^equ^ only.

^§^ Resolved within 12 h (oedema in the “Active Substance Group” persisted for several days).

Biological safety of iNSA/E6E7^equ^ and iNSB/E6E7^equ^ was assessed by repeated RT/PCR and plaque assays from extracts of nasal swabs and faeces (RT/PCR only) collected on days 1, 3, 5, 8, 10 and 12.

To extract total RNA from nasal secretions, swabs were centrifuged at high speed for 5 minutes. Then RNA was isolated from these nasal secretions by using a Viral RNA Mini Kit (Qiagen) in line with manufacturer’s instructions. RNA isolation from faeces was carried out using a ZR Soil/Fecal RNA microPrep^TM^ (Zymo Research, Irvine, CA, USA) according to manufacturer’s recommendations. RT/PCR positive controls consisted of total RNA extracted from nasal swab or faeces aliquots spiked with iNSA/E6E7^equ^ or iNSB/E6E7^equ^. Plasmids harbouring the iNSA- E6E7 or iNSB-E6E7 sequence served as PCR positive controls. RT/PCR was performed as described above. No-reverse transcriptase (no enzyme; -RT) controls were included in all reactions.

For the plaque assays, Vero cells were seeded at a density of 25,000 cells/cm^2^ in OptiPro 2% Glutamax (Sigma-Aldrich) and incubated for 24 h at 37°C. Then the cells were cultured in infection medium (OptiPro containing 2% Glutamax, 0.5% TrypLE, 250 ng/ml amphotericin B, and 4% Anti-Anti; all from Sigma-Aldrich), overlaid with nasal secretions and incubated for 2 h at 37°C. Then the cells were cultured in plaque medium (OptiPro with 2% Glutamax, 4.4 nmol NaHCO_3_, 1% DMEM, 2% DEAE Dextran Hydrochloride, 1% TrypLE, 500 ng/ml amphotericin B and 2% Anti-Anti; all from Sigma-Aldrich). A 1:1000 dilution of BPV1 virions in medium as well as medium only were used as respective positive and negative controls. Three days post infection, the cytopathic effects were analysed by confocal microscopy.

### Patient study

Based on good general health condition and the informed owner’s written consent, 30 sarcoid-affected horses (15 mares and 14 geldings; 2–23 years old) were subsequently enrolled in a patient trial. As from March 2019, equine patients consecutively presented at the Vetmeduni Equine University Hospital. Following an owner interview and thorough clinical examination of the horse, its signalment, basic clinical parameters, as well as sarcoid disease specifications were recorded. The latter included the number, type and location of the lesions, maximum tumour diameters as determined with callipers and the disease history (disease onset, previous treatment and tumour recurrence) (Tables 1 and 3). Based on these parameters, a sarcoid grading system was developed: disease was graded as mild (single sarcoid of mild occult, verrucous, or nodular type/several occult sarcoids), moderate (single sarcoid of nodular or fibroblastic type/single mixed sarcoid), or severe (single malevolent sarcoid/multiple sarcoids including nodular, fibroblastic, mixed or malevolent lesions, recurrent sarcoids) (Table 3). The intralesional presence of BPV infection and BPV types were assessed by BPV1/2 E5 PCR [49] followed by bidirectional amplicon sequencing (Eurofins).

**Fig 2:**
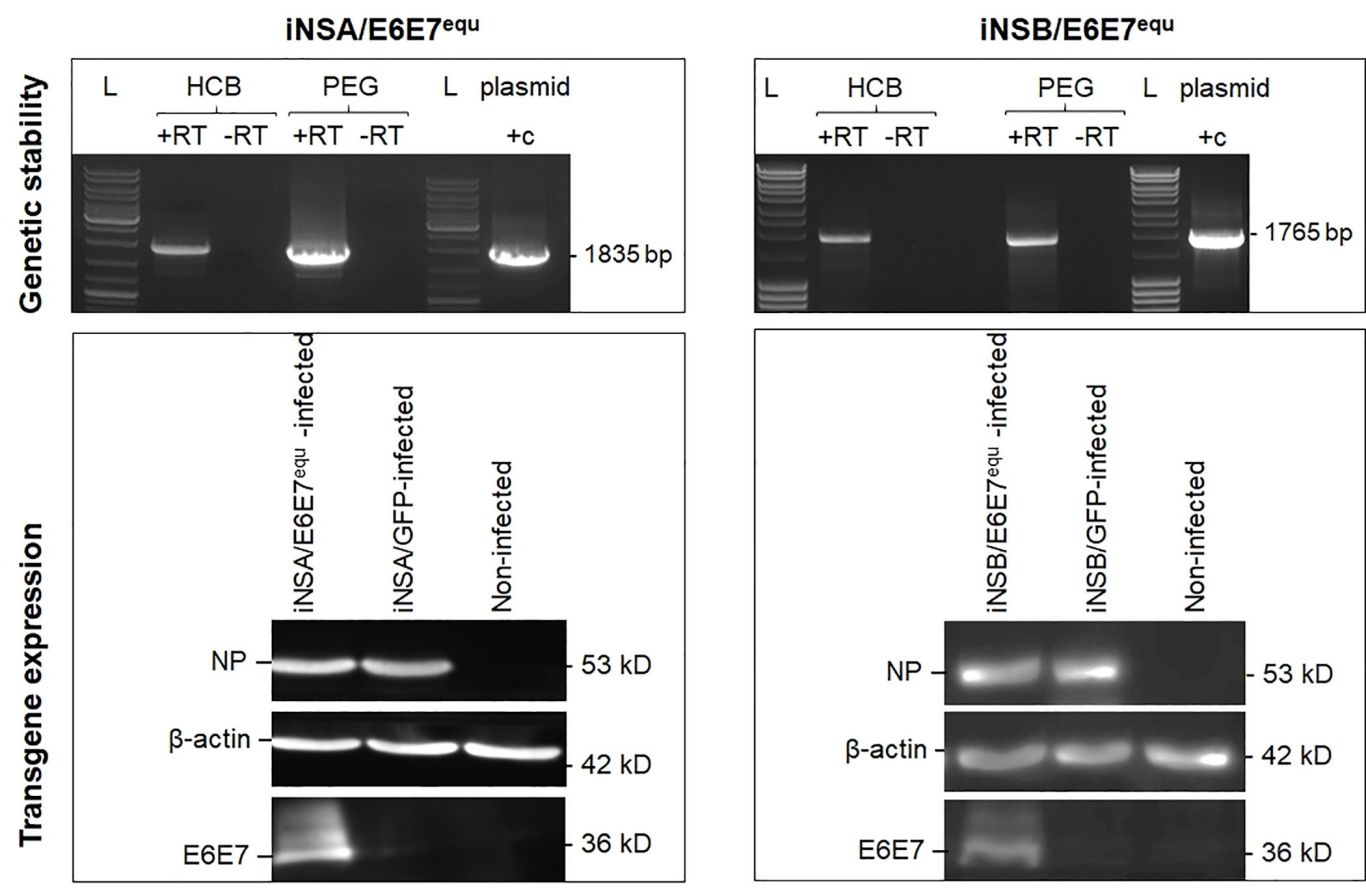
iNSA/E6E7^equ^ and iNSB/E6E7^equ^ were genetically stable and expressed shuffled E6/E7 protein. Top: Transgene stability in the iNS1 reading frame was demonstrated by RT-PCR from total RNA after five passages of iNSA/E6E7^equ^ and iNSB/E6E7^equ^ in Vero cells. Bottom: Western blot analysis of lysates of equine primary palatal fibroblasts (eqPALF) infected with iNSA/E6E7^equ^ (left) and iNSB/E6E7^equ^ (right) revealed stable influenza virus transgene (E6E7) expression.

Treatment was carried out at the BSL-2 facility and generally consisted of repeated intratumoural injections with 500 μl of 9 log FFU of iNSA/E6E7^equ^ or iNSB/E6E7^equ^ in SNH buffer per lesion. In horses bearing more than one sarcoid, at least one tumour was left untreated. The decision as to which tumour(s) should be injected was based on the clinical type (severe types were preferentially injected), the accessibility of the tumour in the standing horse and the compliance of the animal. In a few cases, animals were sedated by intravenous (i.v.) injection with Detomidine (0.01–0.04 mg/kg; Equidor, Richter Pharma AG, Wels, Austria) and Butorphanol (0.02–0.05 mg/kg; Alvegesic, Alvetra und Werfft GmbH, Neufeld an der Leitha, Austria) before treatment. Two different time schedules were implemented: Schedule 1 involved the first eight patients and consisted of intratumoural injections of iNSA/E6E7^equ^ on days 0, 2 and 4, and of iNSB/E6E7^equ^ on days 7, 9 and 10. Schedule 2 involved the next 21 patients and consisted of intratumoural injections of iNSA/E6E7^equ^ and/or iNSB/E6E7^equ^ on days 0, 28 and 86 (Table 3). Time schedule 2 could not be respected in case of three patients (Table 3; indicated by ^§^) that were unable to present at the Vetmeduni in time due to SARS-CoV2-related travel restrictions (Table 2). Several horses that stopped responding following completion of basic treatment received one or two additional booster injections with 9 log iNSA/E6E7^equ^ or iNSB/E6E7^equ^/injection, as indicated (Table 2). All horses were closely monitored for local and systemic adverse effects as described above (safety trial). Tumour diameters were assessed in monthly intervals using callipers and by photography (Fig 2). In addition, patient owners gave feedback on a regular basis. In two cases (BEG, ZAC) showing complete sarcoid regression, it was possible to collect sample material (crusts, hair roots) from previous tumour sites during the last visit. Following DNA extraction from this material, BPV1/2 E5 PCR was carried out. PCR-compatible quality of DNA extracts was confirmed by β-actin PCR.

**Table 2:** Information on sarcoid-bearing horses enrolled in the patient study.

Horse	Age	Sex	Breed	Sarcoid location(s)	Previous treatment
ROS	13	m	WB	cheek, chest, inner thigh, udder	SU, LC, AI
LAW	13	g	WB	ears, chest, both axillae	SU, LC, AI
GOL	9	m	HF	axilla, inner thigh	none
CRS	15	g	QH	chest, flank, abdomen, inner thighs, prepuce	SU
EVS	14	m	HF	cheek, knee, prepuce	SU, AI
FLO	10	g	WB	cheek, inner thigh, prepuce	LC
SAL	19	g	TB	elbow, abdomen, inner thigh	SU, LC
ANC	12	m	WB	axilla, abdomen, inner thigh	none
STF	7	m	WB	axilla	none
DAV	14	g	WB	inner thigh	none
FIN	22	m	IS	muzzle, ears, chest, axilla, anogenital region	SU
MEM	17	g	WB	left, inner thigh	SU, LC, AI
BEG	7	m	PT	periocular region	SU
SCH	2	m	QH	throat, chest, axilla, thorax, abdomen, thigh	none
COM^§^	7	g	WB	ear, throat, axilla, abdomen, inner thigh	none
SLH^§^	8	g	QH	axilla, inner thigh, prepuce	SU
AVE^§^	16	g	HF	inner thigh, genital region	none
SAV	6	g	NO	prepuce	none
FED	10	g	WB	eye lid, neck, inner thigh, abdomen, prepuce	none
ALB	11	m	WB	abdomen	SU
CIN	9	m	HF	abdomen, udder	none
EDW	5	g	WB	periocular region, chest, abdomen	none
CHI	20	g	HF	axilla, inner thigh	SU, LC
SHA	16	m	TB	inner thigh, udder region	SU
ZAC	9	g	NO	upper eye lid	none
LAI	10	m	WB	ear	none
LAN	6	m	WB	periocular region	none
ELA	23	m	HF	abdomen	none
FIA	8	m	WB	ear, axilla, inner thighs	none

m: mare; g: gelding; WB: Warmblood, TB: Thoroughbred; HF: Haflinger; QH: Quarter horse; IS: Icelandic horse; NO: Noriker; SU: surgery; LC: local chemotherapy; AI: auto-implantation

### Ethical approval

All animal studies were carried out with the owners written consent following approval by the institutional ethics and animal welfare committee and the national authority according to §§ 26ff of the Animal Experiments Act, "Tierversuchsgesetz 2012 -TVG 2012" under authorization numbers: 68.205/0132-WF/V/3b/2017, 68.205/0096-V/3b/2018, 68.205/0219-V/3b/2018, 68.205/0103-V/3b/2019, 68.205/0186-V/3b/2019, 2020–0.827.362.

## Results

### iNSA/E6E7^equ^ and iNSB/E6E7^equ^ are genetically stable and express E6/E7 transgenes

iNSA and iNSB influenza viruses harbouring shuffled BPV1 E6 and E7 coding sequences were obtained by genetic engineering. To test for the genetic stability of the fusion of the first 106 codons of NS1 with the E6 and E7 sequences, the viruses were passaged in Vero cells for five rounds. Then total RNA was isolated and screened for the presence of iNSA-E6E7 and iNSB-E6E7 transcripts by RT/PCR. The reactions yielded amplification products of expected size for iNSA/E6E7^equ^ and iNSB/E6E7^equ^ as shown in Fig 2 (top), thus confirming the genetic stability of both virus types.

Transgene expression in infected eqPALF after five passages was verified by Western blot using primary antibodies specific for E6E7-, NP- or beta actin. INSA/E6E7^equ^- and iNSB/E6E7^equ^-infected cells tested positive for E6E7, NP and beta actin expression. EqPALF cells infected with iNSA and iNSB viruses harbouring the ORF of green fluorescent protein (iNSA/GFP; iNSB/GFP) and non-infected EqPALF included as controls tested positive for NP and beta actin, and beta actin only, thus confirming the accuracy of the experiment (Fig 2, bottom).

### Intralesional injection of horses with INSA/E6E7^equ^ and iNSB/E6E7^equ^ is well tolerated

**Fig 3:**
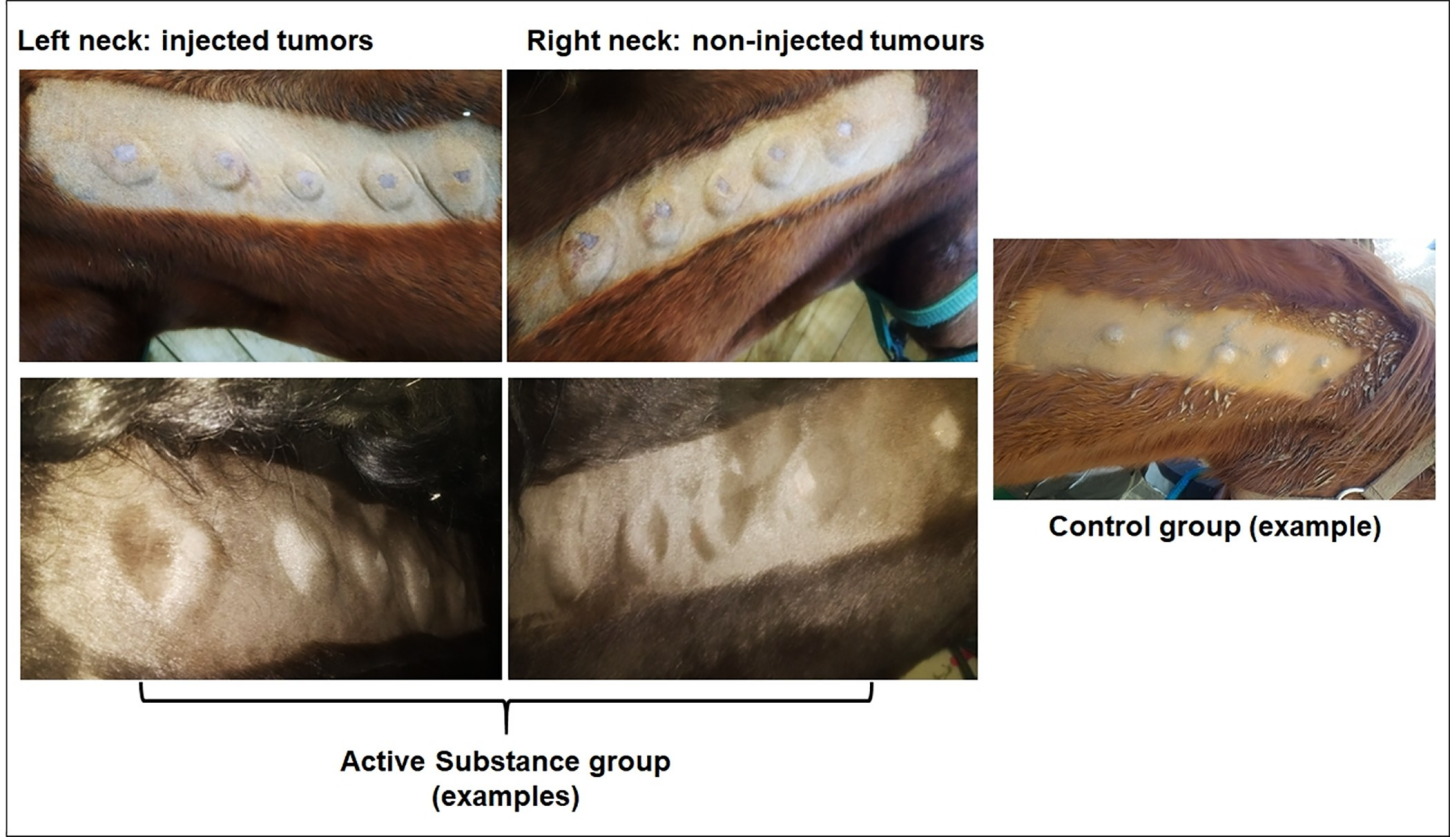
Injection of pseudo-sarcoids with iNSA/E6E7^equ^ and iNSB/E6E7^equ^ induced a systemic immune response. Injection of pseudo-sarcoids with iNSA/E6E7^equ^ or iNSB/E6E7^equ^ (“active substance”) on the left side of the neck induced oedema at the injection sites, and importantly, also at the non-injected tumour sites on the right side of the neck. Intralesional administration of SNH-buffer at the left side of the neck of control horses had no apparent effect, except for short-lived oedema in one horse (not shown). Pseudo-sarcoids on the right side of the neck of control horses remained unchanged.

Twelve horses bearing 2 x 5 experimentally BPV1 virion-induced pseudo-sarcoids on the left and right side of the neck were randomly assigned to an “active substance” (AS; n = 6) or a control (C; n = 6) group. Tumours on the left side of the neck were injected thrice with INSA/E6E7^equ^ and then thrice with iNSB/E6E7^equ^ (AS group) within two weeks, or with SNH buffer in an analogous manner (C group). The trial was blinded for co-authors E.K. Hainisch and A. Rümmele carrying out the injections and the monitoring. In both groups, tumours on the right side of the neck were left untouched. Monitoring of horses revealed no alterations regarding behavioural parameters, respiratory rates and heart rates. As shown in Table 1, 4/6 horses of the AS group developed transient fever after the first injection with INSA/E6E7^equ^, which ranged between 38.4°C and 39.5°C (the physiological body temperature of horses is ≤ 38°C) and persisted for a maximum of 24 hours. All horses of the AS group developed oedema at the injected tumour sites, and also at the non-injected tumour sites on the right side of the neck, as exemplarily shown in Fig 3. All pseudo-sarcoids regressed within several weeks, as anticipated. Regarding the speed of pseudo-sarcoid regression, no difference between the AS group and the C group was noted (not shown).

### INSA/E6E7^equ^ and iNSB/E6E7^equ^ are safe

**Fig 4:**
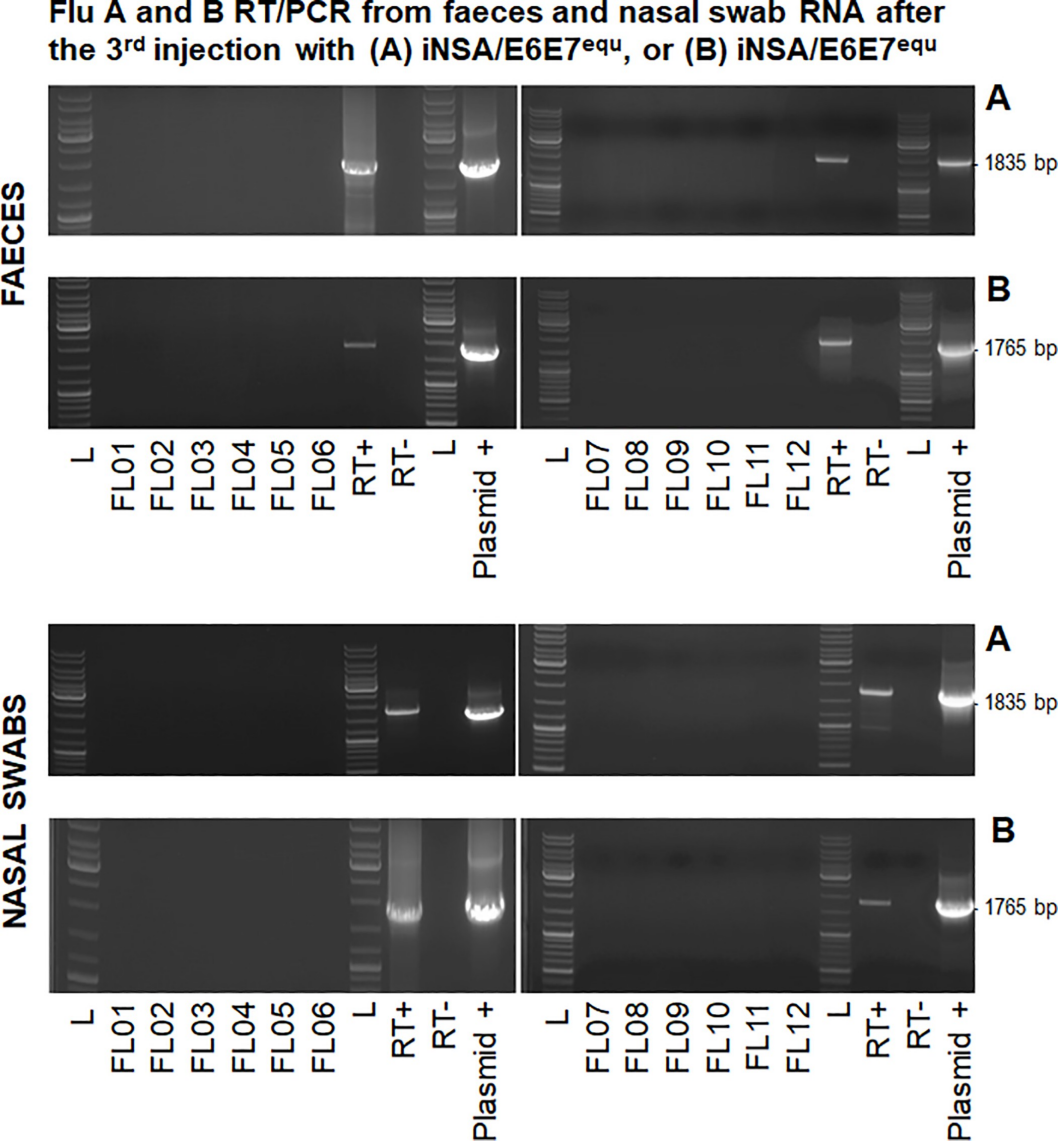
iNSA/E6E7^equ^ and iNSB/E6E7^equ^ were biologically safe. Top: Faeces and nasal swabs collected after each intralesional injection tested negative by RT/PCR, whereas controls yielded anticipated results. L: GeneRuler DNA Ladder mix (ThermoScientific). Bottom: Negative RT/PCR results obtained for nasal swabs were confirmed by plaque assay thus providing additional evidence for the biological safety of iNSA/E6E7^equ^ and iNSB/E6E7^equ^.

Biological safety of both viruses was assumed due to the partial NS1 deletion and intratumoural administration. To confirm this assumption, nasal swabs and fresh faeces were collected after each virus administration during the safety study and assessed for virus shedding. No iNSA/E6E7^equ^ or iNSB/E6E7^equ^ were detected from nasal swabs or faeces by RT-PCR, as shown in Fig 4 (top). In addition, nasal swabs tested negative for both viruses by plaque assay (Fig 4, bottom). Included RT+, RT- and PCR+ controls confirmed the authenticity of the results.

### INSA/E6E7^equ^ and iNSB/E6E7^equ^ can induce sarcoid regression even in severe cases

From the 29 sarcoid-bearing horses enrolled in the patient study, 19 presented with severe (S), five with moderate (M), and five with mild (m) disease (Table 3). Fifteen patients had a history of previous, ineffective treatment(s) by surgery (SU), local chemotherapy (LC), and/or autologous implantation (AI) (Table 2).

**Table 3:** Response to treatment.

Horse	Grade	Months										
		1	2	3	4	5	6	7	8	9	10	11–19	20
**iNSA/E6E7**^**equ**^ ***on d0*, *d2*, *d4*, iNSB/E6E7**^**equ**^ ***on d7*, *d9*, *d11 (n = 8)***													
ROS	S	➘	➘	➘	➚	➚	**rpt**	➚	➚			**B**_**a**_	**SU**
LAW	S	➘	➘	➘	➚	➚	**rpt**	➘	➘	➘	➚	Put to sleep	
GOL	S	➞	➞	➞	➞	➞	➞	end					
CRS	S	➞	➞	➞	➞	➞	➞	end					
EVS	S	➞	➞	➚	➚	➚	➚	end					
FLO	S	➘	➘	➘	➘	➘	➘	➘	➘	➘	➘	**CR**	
SAL	M	➞	➞	➘	➘	➘	➘	➘	➘	➘	**CR**		
ANC	S	➘	➘	➘	➘	➘	➘	➘	➘	➘	➘	**CR**	
**iNSA/E6E7**^**equ**^ ***on d0*, *d28*, *d86 (n = 10)***													
STF	m	➘	➘	➘	➘	➘	➘	➘	**CR**				
DAV	m	➘	➘	➘	➘	➘	➘	**CR**					
FIN	S	➞	➞	➚	➚	➚	➚	end					
MEM	M	➘	➘	➘	➘	➘	**CR**						
BEG	S	➚	➚	➚	➘	➘	➘	➘	➘	**CR**			
SCH	S	➞	➚	➚	➚	➚	➚	end					
COM^§^	S	➞	➞	➞	➞	➚	➞	➘	➘	**B**_**a**_➘	➘		
SLH^§^	M	➘	➘	➘	➘	➘	➘	➘	**CR**				
AVE^§^	M	➘	➘	➘	➘	➘	➘	sold					
SAV	M	➞	➞	➞	➘	➘							
**iNSA/E6E7**^**equ**^ ***on d0*, *d28*, iNSB/E6E7**^**equ**^ ***on d86 (n = 4)***													
FED	S	➞	➞	➞	➞	➘	➘	➘	**B**_**b**_	➘	**B**_**a**_➘		
ALB	m	➞	➞	➘	➘	➘	**CR**						
CIN	S	➘	➞	➞	➞	➚							
EDW	S	➘	➘	➘	➘	➘							
**iNSB/E6E7**^**equ**^ ***on d0*, *d28*, *d86 (n = 3)***													
CHI	S	➞	➘	➘	➘	one tumour regresses, second daily “cleaned” with alcohol by owner -> **SU**							
SHA	S	➞	➞	➞	➚	➚	➚	**SU**					
ZAC	S	➘	➘	➘	➘	➘	➘	**CR**					
**iNSB/E6E7**^**equ**^ ***on d0*, *d28*, iNSA/E6E7**^**equ**^ ***on d86 (n = 4)***													
LAI	m	➘	➘	➘	➘								
LNO	m	➘	➘	➘									
ELA	S	➘	➞	➞	➘								
FIA	S	➞	➞	➞	➘								

S: severe; M: moderate; m: mild; rpt: scheme repeated; B_a_, B_b_: additional boost with iNSB/E6E7^equ^ or iNSB/E6E7^equ^; CR: complete regression; SU: Surgery

**Fig 5:**
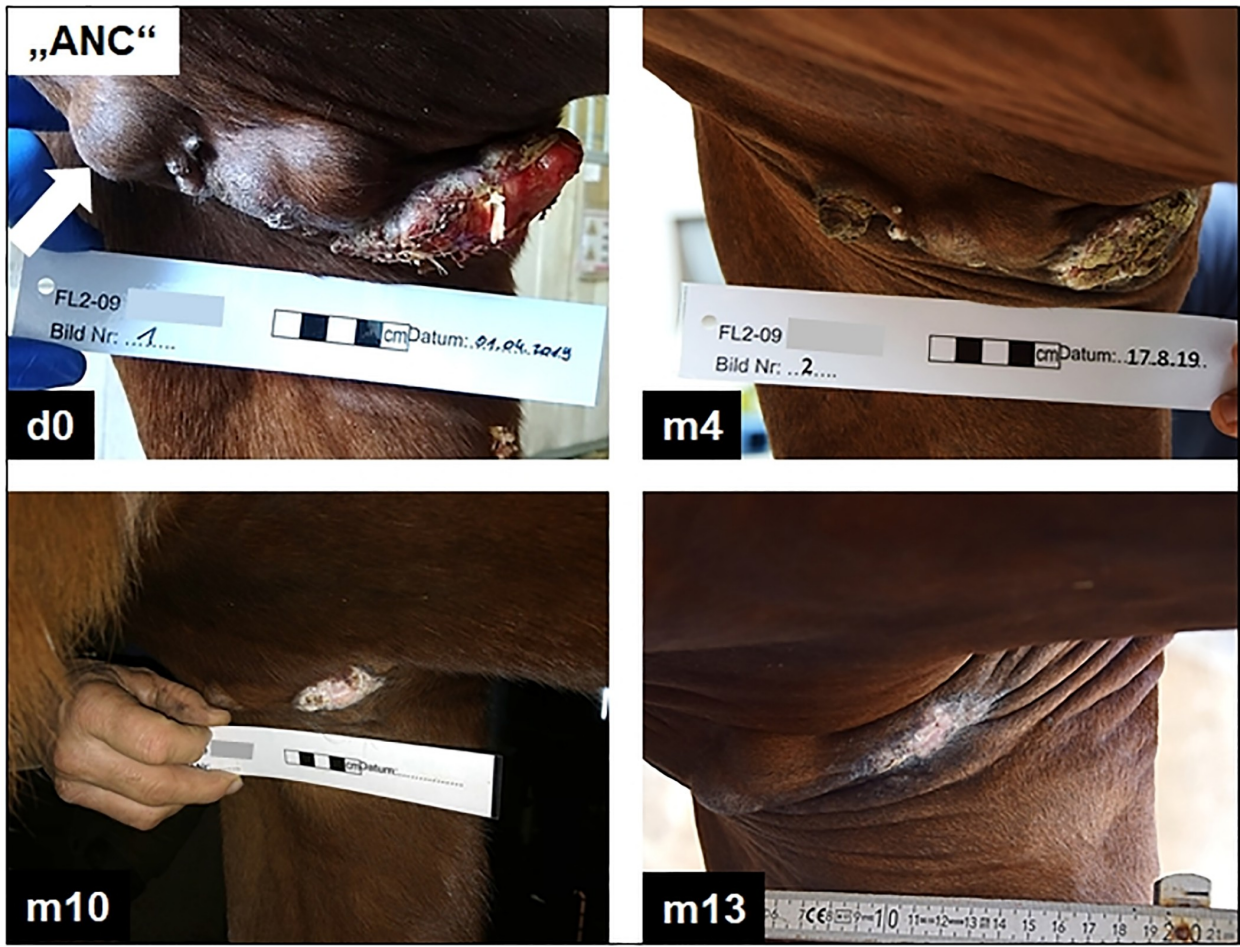
Intratumoural injection with iNSA/E6E7^equ^ and/or iNSB/E6E7^equ^ led to tumour regression even in equine patients with severe disease. A mare (ANC) with multiple, severe-type sarcoids affecting the right axilla, abdomen and inner thigh responded to intratumoural therapy. Injection of a single lesion with iNSA/E6E7^equ^ on days 0, 2 and 4, and iNSB/E6E7^equ^ on days 7, 9 and 11 by (white arrow indicates injection site) led to complete regression of the injected and all non-injected sarcoids within 13 months as exemplarily shown for the right axilla. d0: day 0; m4: month 4; m10: month 10; m13: month 13.

The first eight patients received 2 x 3 intratumoural injections with iNSA/E6E7^equ^ and iNSB/E6E7^equ^ within two weeks and were permanently kept within the BSL-2 facility for this purpose. Two horses with severe disease (ROS, LAW) initially responded to the therapy as revealed by dramatic tumour regression within the first three months after treatment. However, the sarcoids subsequently resumed growth. Another treatment round in month 6 had a transient therapeutic effect in patient LAW, whereas ROS failed to respond (Table 3). Three horses with severe disease (GOL, CRS, EVS) failed to clinically respond to treatment, albeit by stable disease. Consequently, treatment was discontinued. In two horses (FLO, ANC) with severe and one patient (SAL) with moderate disease, treatment resulted in complete regression (as defined as: “disappearance of all target lesions” according to [51]) of injected and non-injected sarcoids within ten to thirteen months (Table 3). Fig 5 exemplary shows the healing process for patient ANC.

**Fig 6:**
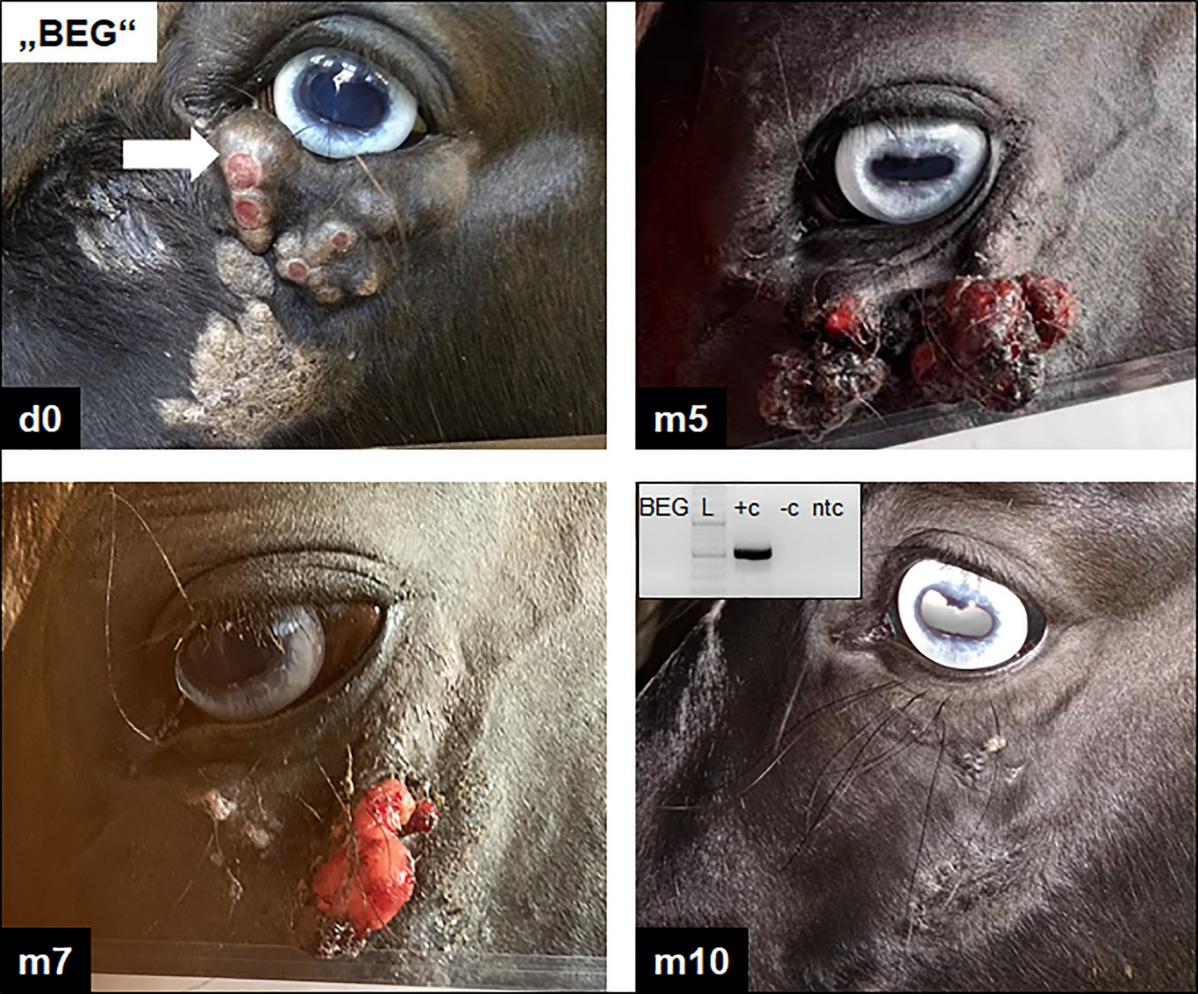
Intratumoural injection with iNSA/E6E7^equ^ *on d0*, *d28*, *d86* led to tumour regression and no BPV1 DNA was detectable anymore from the tumour site. A mare (BEG) suffering from periocular mixed sarcoids (fibroblastic, nodular and verrucous components) shows complete tumour regression 10 months after intratumoural injections (white arrow indicates injection site) with iNSA/E6E7^equ^ on days 0, 28 and 86. Importantly, BPV1 E5 PCR from DNA of crusts collected from scarified skin (month 10) scored negative (see inserted gel photo; BEG: patient DNA; L: GeneRuler DNA Ladder mix [ThermoScientific]; +c: sarcoid DNA as positive control: -c: equine intact skin DNA as negative control; ntc: sterile water as no-template control); d0: day 0; m5: month 5; m7: month 7; m10: month 10.

**Fig 7:**
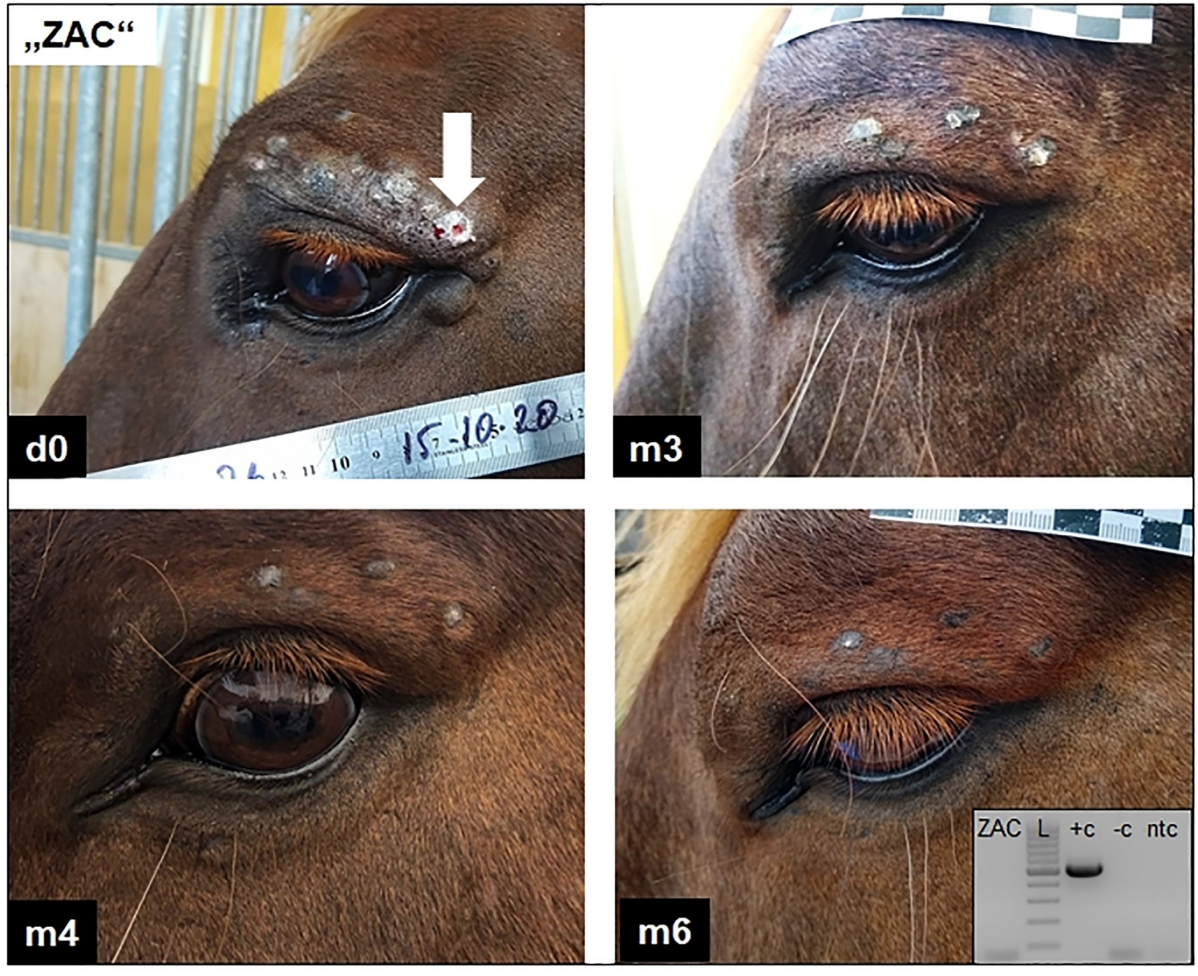
Intratumoural injection with iNSB/E6E7^equ^ *on d0*, *d28*, *and d86* led to tumour regression and no BPV1 DNA was detectable anymore from the tumour site. A gelding (ZAC) suffering from periocular mixed sarcoids (fibroblastic, nodular and verrucous components) shows complete tumour regression 6 months after intratumoural injections (white arrow indicates injection site) with iNSB/E6E7^equ^ on days 0, 28 and 86. BPV1 E5 PCR from DNA of hair roots collected from scarified skin during the last visit (month 6) also scored negative (see inserted gel photo; ZAC: patient DNA; L: GeneRuler DNA Ladder mix [ThermoScientific]; +c: sarcoid DNA as positive control: -c: equine intact skin DNA as negative control; ntc: sterile water as no-template control); d0: day 0; m3: month 3; m4: month 4; m6: month 6.

To explore the possibility of reducing the stabling time at the BSL-2 facility and further improving treatment efficacy, all further patients received intratumoural injections on days 0, 28 and 86. According to this time schedule, the next 10 horses (STF—SAV) were exclusively treated with iNSA/E6E7^equ^. From these, two severely affected individuals failed to respond to treatment (Table 3). In two horses with mild, two with moderate and one with severe disease, treatment induced complete sarcoid regression. In one severely and two moderately affected patients, sarcoid regression is ongoing and under observation (Table 3). Comparable results were obtained in sarcoid patients treated with iNSA/E6E7^equ^ on days 0 and 28 and iNSB/E6E7^equ^ on d86 (n = 4; 3/4 responders to therapy), iNSB/E6E7^equ^ only on days 0, 28, and 86 (n = 3; 2/3 responders) or iNSB/E6E7^equ^ on days 0 and 28 and iNSA/E6E7^equ^ on day 86 (n = 4; 4/4 responders). An overview on current treatment results is given in Table 4. Independently of the treatment scheme, complete sarcoid regression was achieved in 10 patients (four severe, three moderate and three mild cases) (Figs 6 and 7). In another 10 patients (six severe, two moderate and two mild cases), injected (and non-injected) sarcoids continue to regress, as documented by monitoring (not shown).

**Table 4:** Overview on current treatment results (May 2021).

Effect of treatment	Number of patients	% of patients
No sarcoid regression	6	20.6
Transient sarcoid regression	3	10.4
Complete sarcoid regression	10	34.5
Sarcoid regression still ongoing	10	34.5
Total	29	100

### INSA/E6E7^equ^ and iNSB/E6E7^equ^ have an impact on BPV infection levels

In two severe cases, where complete regression of mixed, periocular sarcoids could be achieved by intratumoural injections with iNSA/E6E7^equ^ on days 0, 28 and 86 (BEG), or iNSB/E6E7^equ^ on days 0, 28 and 86 (ZAC), crusts (BEG) and hair roots (ZAC) collected from previous tumour sites during the last visit were subjected to DNA extraction and subsequent BPV1/2 E5 PCR. Both samples tested negative (Figs 6 and 7). The PCR-compatible quality of DNA extracts was confirmed by β-actin PCR (not shown). Sarcoid DNA, equine skin DNA and sterile water included in the BPV1/2 E5 amplification reactions as positive, negative, and no-template controls yielded expected results, thus emphasising the technical accuracy of the experiment (Figs 6 and 7).

## Discussion

Sarcoids are BPV-induced skin tumours in equids that do not metastasise. However, they can affect a considerable portion of the skin and progress to such an extent that they still constitute the major dermatological reason for euthanasia [4]. This is also due to the fact that sarcoids tend to resist therapy and to recrudesce in a more progressive form following ineffective treatment. Accidental trauma is an additional factor promoting the progression of mild-type to multiple severe-type lesions [5]. Current therapeutic approaches focus on the eradication of tumour masses, be it by surgical (laser-sustained) excision, local application of chemotherapeutics such as imiquimod, cis-platin or mitomycin or exposure to extreme heat (thermotherapy) or cold (cryotherapy), with varying success [8]. Radiotherapy has proven highly effective in sarcoid therapy. However, this treatment approach is no option for the majority of sarcoid patients as only few veterinary hospitals worldwide offer this possibility [8].

Human high-risk PVs (hrHPVs) cause distressing, potentially lethal cancer diseases in humans. In recent years, considerable efforts have been made to develop immunotherapeutic vaccines that induce or enhance a hrHPV-specific immune response and by this, promote the killing of infected tumour cells [33]. In equine sarcoid management, the only immunotherapeutic approach known so far consists of the autologous re-implantation of minced, cryo-inactivated sarcoid tissue [52, 53]. This method is effective in a subset of cases, indicating that it can re-instruct the immune system to recognize PV and tumour-associated antigens. However, no immunological data are available so far. Moreover, the method cannot be standardised, notably regarding delivered antigen concentrations, and abscessation following autologous implantation is also reported (Espy, 2008; Rothacker et al., 2015; E.K. Hainisch, personal communication). Based on promising data demonstrating the antitumour potential of NS1-deleted influenza A viruses co-expressing HPV type 16 E6E7 in a murine TC1 tumour model [43, 45], we have generated partially NS1-deleted influenza A and B viruses co-expressing shuffled BPV1 E6E7 (iNSA/E6E7^equ^ and iNSB/E6E7^equ^) for sarcoid immunotherapy. Previous studies have shown that conservation of the first 106 C-terminal amino acids of the NS1 gene (iNS) confers sufficient live attenuation but permits efficient viral replication also in IFN-competent tumour cells. Viral replication is important for the expression of tumour antigens (unpublished). In accordance with these data, we could show that BPV1 E6E7 is stably co-expressed by iNSA/E6E7^equ^ and iNSB/E6E7^equ^ in infected equine primary palatal fibroblasts.

Previous in vivo studies have shown that (transgene-expressing) NS1-deleted influenza A and B viruses are well tolerated when applied intranasally, subcutaneously or into the muscle [38, 40, 42, 43, 45, 54]. Similarly, injection of pseudo-sarcoids (five per AS horse) with 9 log FFU of iNSA/E6E7^equ^ and then iNSB/E6E7^equ^/lesion every other day was well tolerated. The immunogenicity of both viruses was clinically reflected by transient fever in four of six, and by oedema at the injection sites in all six treated individuals. Importantly, virus-treated horses also developed oedema at all non-injected tumour sites on the right side of the neck. This finding clearly indicates that iNSA/E6E7^equ^ and iNSB/E6E7^equ^ induced a systemic immune response. This assumption was corroborated in the patient study. Experimental pseudo-sarcoids differ from natural sarcoids in that they regress spontaneously [25]. Virus-, mock- and non-injected pseudo-sarcoids showed a similar regression speed. This finding was anticipated on the grounds that spontaneous pseudo-sarcoid regression driven by a highly alerted immune system occurs within weeks, leaving a very narrow observation window.

The patient study involved a total of 29 sarcoid-affected horses of different age and breed, and all horses received the active substance. It may be argued that a group of untreated sarcoid patients should have been included as control. We opted against this possibility for the following reasons: First, sarcoids are usually persistent tumours that do not regress spontaneously [5]. Second, refusing treatment to, or mock-treating sarcoid-patients is ethically questionable. On the one hand, sarcoids have to be treated as soon as possible according to health and welfare standards, and on the other hand, mock-injection is invasive and the inflicted trauma might promote disease progression [5].

Exclusive or combined intratumoural administration of iNSA/E6E7^equ^ and/or iNSB/E6E7^equ^ resulted in sarcoid regression in 20/29 patients. Intriguingly, regression was not restricted to injected sarcoids but also observed for non-injected lesions, which is consistent with a systemic and sarcoid-specific immune response. In most cases, both time schedules (intratumoural virus administration on days 0, 2, 4, 7, 9 and 11, or on days 0, 28 and 86) led to satisfactory results. Given that the first schedule requires permanent stabling of horses at a BSL-2 facility for more than two weeks, whereas the second schedule only necessitates three stays overnight, the latter has been adopted as standard procedure for welfare reasons.

We also investigated whether a combination of iNSA/E6E7^equ^ and iNSB/E6E7^equ^ for prime and boost treatment is necessary to achieve a maximum therapeutic effect. Whereas iNSA/E6E7^equ^ and iNSB/E6E7^equ^ contain identical E6 and E7 antigens, the vector backbones stem from antigenically completely different influenza viruses (A and B). The rationale behind the combined approach was that the use of antigenically different vectors may help overcome preexisting immunity to the vector after several treatments, that may reduce the effectiveness of boost treatments. While sarcoids in several horses (e.g. ELA, FIA, FED) started regressing just after the heterologous boost at day 86, three horses treated solely with homologous vector also began to regress after the third treatment (SAV, BEG, COM). Therefore, we will need to address this issue in larger numbers of patients per group as to be able to provide conclusive data. However, our current findings suggest that exclusive repeated treatment with iNSA/E6E7^equ^ or iNSB/E6E7^equ^ is as efficient as the successive administration of both viruses. In this context, the severity and persistence of the disease as well as a history of previous unsuccessful therapeutic interventions seemed to be more decisive for the therapeutic outcome than injection schedules and schemes. The six non-responders and the three transiently responding horses all presented with severe disease, i.e. multiple sarcoids of all types, including aggressive fibroblastic sarcoids, at different sites of the body. Five of these patients had a history of unsuccessful treatment.

Prior to treatment, all horses of the patient study underwent a clinical examination leading to sarcoid diagnosis. The latter was confirmed by BPV1/2 PCR from DNA of sarcoid scrapings, which also revealed the exclusive presence of BPV1 in the samples. Following successful therapy of severe, periocular sarcoid disease in two horses, BPV1/2 PCR from crusts or hair roots collected from previous tumour sites scored negative. To our knowledge, this is the first report on a sarcoid therapeutic that is not only reported to induce tumour regression, but has also an impact on BPV1 infection levels. This finding is not completely surprising, as we used an immunotherapeutic approach targeting the causative viral agent underlying the disease. Provided these data can be strengthened by in-depth infection screening of further patients with complete sarcoid regression following iNSA and/or B/E6E7^equ^ therapy, they are absolutely unique. In cases, where clearance from infection could demonstrably be achieved, it might be possible to reach sustainable freedom from disease.

In this study, we did not include the parenteral influenza virus A and B strains as controls that would allow for assessment of the specific therapeutic effect of iNSA/E6E7^equ^ and iNSB/E6E7^equ^. Due to ethical considerations, we have addressed this issue in a HPV16 TC-1 mouse model providing consistent evidence that the antitumor effect was mainly due to the expression of the tumour antigens. In this model, we could also demonstrate that tumour regression was highly dependent on virus dosage, and associated with strong infiltration of tumour-specific CD3^+^ and CD8^+^ T lymphocytes [43] (TM, personal communication). An in-depth study addressing the immunological mechanisms underlying iNSA/E6E7^equ^- and iNSB/E6E7^equ^-induced tumour regression is ongoing.

All tested sarcoids in the patient group were shown to harbour BPV1. This is not surprising, since BPV1 is the prevailing type detected in Central European sarcoid patients. BPV1 and BPV2 are genetically highly homologous PVs, as reflected by an overall sequence identity of 87%. On these grounds, it can be speculated that iNSA/E6E7^equ^ and iNSB/E6E7^equ^ may be also suited for immunotherapy of BPV2-induced sarcoids. Alternatively, therapeutic constructs could be genetically adapted. Further research will help clarify this issue.

The presented findings are very encouraging. Although we plan to further refine the iNSA and/or B/E6E7^equ^ treatment modalities based on awaited immunological data, the strategy has already proven effective in reducing or eliminating the sarcoid burden even in severe cases, and in clearing the BPV1/2 infection that underlies disease development. Although BPV1 and BPV2 on the one hand, and hrHPVs on the other hand, belong to different groups of PVs that considerably differ regarding their respective cell tropism, host spectrum and carcinogenic potential, they all adhere to common principles with respect to their structure and manifold functions. The early oncogenes E5, E6 and E7 of BPV1, BPV2 and hrHPVs transform infected normal cells into neoplastic cells and use similar mechanisms to create a protumoural immune microenvironment [10, 55–58]. Others and our group have shown that IFN-induction mediated by delNS-based viruses leads to activation of different components of the immune system and therefore has the potential to overcome immunosuppression created by tumours [59–63]. On these grounds, and based on previous murine data [43, 45] and herein presented findings, it can be speculated that partially or fully NS1-deleted influenza A and B virus co-expressing hrHPV antigens may also be effective in the management of hrHPV-induced human cancers.

## Supporting information

S1 Raw images (PDF)
