# Supplementary material for: Influenza virus vector iNS1 expressing bovine papillomavirus 1 (BPV1) antigens efficiently induces tumour regression in equine sarcoid patients
Source: PLoS One. 2021 Nov 19;16(11):e0260155. doi: 10.1371/journal.pone.0260155 (PMC8604313; doi:10.1371/journal.pone.0260155)
Supplement: S1 Raw images — (PDF) [file pone.0260155.s001.pdf]

Zur optimalen Anzeige dieses PDF-Portfolios sollte es in Acrobat oder Adobe Reader ab Version X geöffnet werden.

Adobe Reader jetzt herunterladen
